# Elevated Serum Levels of Mannose-Binding Lectin and Diabetic Nephropathy in Type 2 Diabetes

**DOI:** 10.1371/journal.pone.0119699

**Published:** 2015-03-24

**Authors:** Ling-Zhi Guan, Qiang Tong, Jing Xu

**Affiliations:** Department of Endocrinology, Xinqiao Hospital, Third Military Medical University, Chongqing, P. R. China; Oxford University, UNITED KINGDOM

## Abstract

**Objective:**

Inflammation and complement activation initiated by mannose-binding lectin (MBL) may be implicated in the pathogenesis of diabetic vascular complications. We investigated serum MBL levels in type 2 diabetes with diabetic nephropathy (DN) and with persistent normoalbuminuria.

**Method:**

Serum MBL levels were determined in 242 type 2 diabetes with overt nephropathy and 242 type 2 diabetes with persistent normoalbuminuria matched for age, sex, and duration of diabetes, as well as in 100 healthy control subjects. The prediction value of MBL was compared with HbA1c, Hs-CRP and with other known predictors. Multivariate analyses were performed using logistic regression models.

**Results:**

The serum MBL levels were significantly higher in diabetes with DN as compared to with persistent normoalbuminuria (P<0.0001). Multivariate logistic regression analysis adjusted for common factors showed that serum MBL levels≥2950ug/L was an independent indictor of DN (OR=7.55; 95%CI: 3.44–19.04). Based on the ROC curve, the optimal cutoff value of serum MBL levels as an indicator for diagnosis of DN was projected to be 2950ug/L, which yielded a sensitivity of 77.2 % and a specificity of 80.8%, with the area under the curve at 0.809 (95%CI, 0.769—0.848).

**Conclusion:**

Our findings suggested that MBL may be involved in the pathogenesis of DN in type 2 diabetes, and that determination of MBL status might be used to identify patients at increased risk of developing nephropathy complications.

## Introduction

Type 2 diabetes (T2DM) has become a major public health problem in China. In 2009, the age-standardized prevalences of total diabetes and prediabetes were 9.7% and 15.5%, respectively, accounting for 92.4 million adults with diabetes and 148.2 million adults with prediabetes [1]. Diabetic nephropathy (DN) is one of the major complications of type 1 and type 2 diabetes and it is associated with end-stage renal failure, cardiovascular disease, and increases mortality of diabetic patients [2]. Early detection may enable development of specific drugs and early initiation of therapy, thereby postponing/preventing the need for renal replacement therapy. In recent years, accumulated data have emphasized the critical role of inflammation in the pathogenesis of DN. Previous studies had found that expression of cell adhesion molecules, growth factors, and pro-inflammatory cytokines are increased in the renal tissues of diabetic patients, and serum and urinary levels of cytokines and cell adhesion molecules, correlated with albuminuria[3].

Mannose-binding lectin (MBL) is synthesized by hepatocytes and belongs to the family of C-type lectins[4]. Its carbohydrate recognition domains bind in a calcium-dependent manner to patterns of carbohydrate residues found on microorganisms. Functional MBL deficiency occurs in as many as 10% of the normal population, and these individuals may be at increased risk of infections [5]. MBL may aggravate local and systemic inflammation through complement activation [6], and it has been documented that inhibition of the complement cascade both at the level of MBL and further downstream improves outcome in patients with acute myocardial infarction [7].

Inflammation and complement activation initiated by MBL may be implicated in the pathogenesis of diabetes and diabetic vascular complications. Emerging evidence indicates that in some situations MBL may cause inexpedient complement activation and tissue injury through binding to endothelial glycosylations. Megia et al. [8] found that MBL gene polymorphisms are associated with gestational diabetes mellitus. Bouwman et al. [9] reported that MBL serum concentration was significantly higher in new-onset patients with diabetes compared with their siblings matched for high-producing MBL genotypes. Another study suggested that MBL may be involved in the pathogenesis of micro-and macrovascular complications in type 1 diabetes [4].

Previous studies found that in patients with T1DM, high levels of circulating MBL have been associated with the development of DN and the presence of cardiovascular disease [4, 10]. The relationship between MBL levels and DN in patients with T2DM remains unknown. Interestingly, Hansen et al. [5] reported that in patients with T2DM, measurements of MBL alone or in combination with CRP can provide prognostic information on mortality and the development of albuminuria. Currently, no data are available on the role of MBL in the progression of DN in Chinese patients with T2DM. In this study, we therefore evaluated serum MBL levels in T2DM with DN and with persistent normoalbuminuria.

## Method

The subjects were T2DM patients who were hospitalized at XinQiao Hospital, Third Military Medical University during the period from May 2012 to June 2014. All patients with long-standing T2DM and DN were recruited for this study. A total of 242 patients with DN and 242 patients with persistent normoalbuminuria (UAE<30 mg/24 h), matched for sex, age, and duration of diabetes were recruited for this study. Exclusion criteria were: decreased level of consciousness, severe aphasia or dysarthria, liver insufficiency, metabolic abnormalities and significant acute medical illness (e.g. infection, autoimmune disease, malignant tumor).

DN was diagnosed clinically based on the following criteria: persistent albuminuria >300 mg/24 h in at least two of three consecutive 24-hurine collections, presence of retinopathy, and no evidence of other kidney or renal tract disease [4]. Diabetes was defined as self-report of a previous diagnosis of the disease by a clinician (excluding gestational diabetes mellitus) or hemoglobin A1c of 6.5% or greater (American Diabetes Association’s new diagnostic criterion for undiagnosed diabetes)[11]. Diabetic retinopathy (DR) was defined as the presence of 1 or more retinal microaneurysms or retinal blot hemorrhages with or without more severe lesions (hard exudates, soft exudates, intraretinal microvascular abnormalities, venous beading, retinal new vessels, preretinal and vitreous hemorrhage, and fibroproliferans) using the Early Treatment Diabetic Retinopathy Study (ETDRS) grading standards. DR severity was categorized as non-proliferative diabetic retinopathy (NPDR; level 20 through level 53) and proliferative diabetic retinopathy (PDR; level≥60). A group of 100 age-matched healthy subjects served as control subjects. The study followed the tenets of the Declaration of Helsinki and was approved by the Institute ethics committee of XinQiao Hospital of Third Military Medical University, with written informed consent obtained from each participant.

At admission, we requested individual participant data regarding presence and severity of DN, DR, age, sex, ethnicity, diabetes duration, hemoglobin A1c (HbA1c), systolic and diastolic blood pressure, cigarette smoking status, body mass index (BMI), and current use of diabetes, antihypertensive, and lipid-lowering medications.

All investigations were performed in the morning after an overnight fast. Venous blood was drawn with minimal stasis from an antecubital vein. Clotted blood was centrifuged within 1 h and serum stored at _80°C. The Urinary Albumin Excretion (UAE) was determined in 24-hour urine collections by enzyme-linked immunosorbent assay thereafter (sensitivity, 0.001 mg/L; CV, 4.5–7.6%). HbA1c was measured by high-performance liquid chromatography (HLC-723 G7; TOSHO, Japan) with a normal range of 4–6%. Other biochemical parameters were assessed using OLYMPUS AU2700 (OLYMPUS, Tokyo, Japan). MBL was measured by time-resolved immune-fluorometricassay on serum samples. Microwells coated with anti-MBL antibody were incubated with dilutions of patient serum, were developed with europium-labelled anti-MBL antibody, and europium was quantified with time-resolved fluorometric assay (Baoman Biological Technology Co., Ltd, Shanghai, China). The detection limit was 1.5ug/L. The standard concentrations in these kits range from 1.5 to 100ug/L, providing a range of 150–10000ug/L at 1/100 dilution. The coefficients of variation (CV) for the intra-and inter-assay reproducibility are 4.0–5.5% and 6.1–8.5%, respectively. For all measurements, levels that were not detectable were considered to have a value equal to the lower limit of detection of the assay.

Results are expressed as percentages for categorical variables and as medians (interquartile ranges, IQRs) for the continuous variables. Univariate data on demographic and clinical features were compared by Mann-Whitney U-test or Chi-Square test as appropriate. Correlations among continuous variables were assessed by the Spearman rank-correlation coefficient. To investigate whether MBL allows predicting of DN in diabetes different statistical methods were used. First, the relation of MBL with the DN was investigated with the use of logistic regression models. We used crude models and multivariate models adjusted for all significant predictors and report odds ratios (ORs). For multivariate analysis, we included confounders, known risk factors, and other predictors as assessed in univariate analysis. Second, receiver operating characteristic curves (ROC) was used to test the overall predict accuracy of MBL, and results were reported as area under the curve (AUC). All statistical analysis was performed with SPSS for Windows, version 20.0 (SPSS Inc., Chicago, IL, USA). Statistical significance was defined as p < 0.05.

## Results

### Patient characteristics

There were 242 patients with DN and 242 patients with persistent normoalbuminuria were eligible for the study. The median age of patients included in this study was 65(IQR, 54–77) years and 59.1% were men. The median time of diabetes duration was 12.5 (IQR, 8.0–18.0) years. Basal characteristics of those patients were provided in Table 1.

**Table 1:** Basal characteristic of diabetes patients with DN or normoalbuminuria.

Characteristics	T2DM		
	DN(n = 242)	Normoalbuminuria (n = 242)	P
Age at baseline (IQR, years)	65(54–77)	65(54–77)	NS
Male (%)	59.1	59.1	NS
Diabetes duration (IQR, years)	12.5(8.0–18.0)	12.5(8.0–18.0)	NS
BMI (IQR, kg/m2)	27.1(25.9–30.3)	26.8(25.1–29.6)	NS
Systolic blood pressure (IQR, mmHg)	147(129–159)	126(120–145)	<0.001
Smoking status (%)	50.4	47.5	NS
Intensive glucose treatment (%)	49.6	36.4	0.016
Blood pressure treatment (%)	59.1	37.2	0.008
Use of lipid-lowering medication (%)	45.5	28.9	0.011
Laboratory findings(IQR)			
HbA1c (%)	8.5(7.8–9.8)	7.0(6.4–8.2)	<0.001
UAE(mg/24h)	815(329–2050)	10(5–16)	<0.0001
Serum creatinine (umol/L)	105(77–138)	74(60–85)	<0.001
Total cholesterol (mmol/L)	5.2(4.2–5.9)	4.4(3.7–5.2)	0.021
Hs-CRP(mg/dL)	1.66(0.60–3.18)	0.82(0.44–1.83)	<0.001
MBL(ug/L)	3325(2983–3760)	2470(2105–2942)	<0.0001
Any DR (%)			—
None	—	29.8	
Simple	31.3	53.7	
PDR	68.7	16.5	

Results are expressed as percentages or as medians (IQR); DN, diabetic nephropathy; BMI, body mass index; Hs-CRP, High-sensitivity- C-reactive protein; UAE, Urinary Albumin Excretion; HbA1c, hemoglobin A1c; DR, diabetic retinopathy; PDR, proliferative diabetic retinopathy.

## Main Results

**Fig 1:**
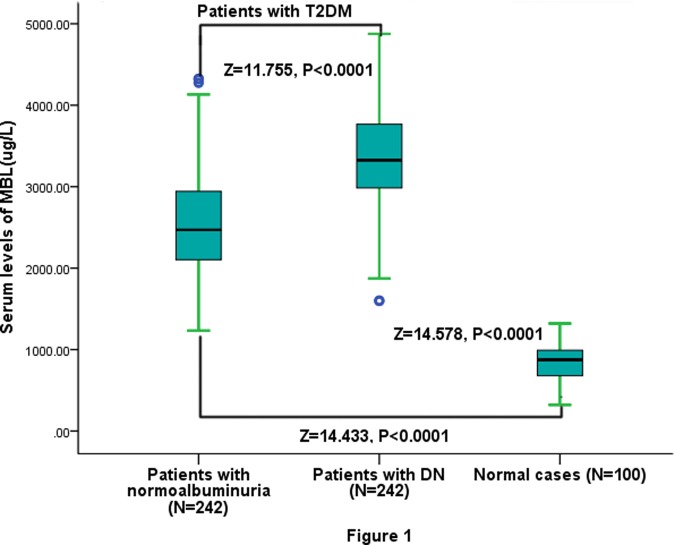
Distribution of serum MBL levels in diabetic patients with diabetic nephropathy (DN) and with persistent normoalbuminuria and normal controls. All data are medians and in-terquartile ranges (IQR). *P* values refer to Mann-Whitney *U* tests for differences between groups.

**Fig 2:**
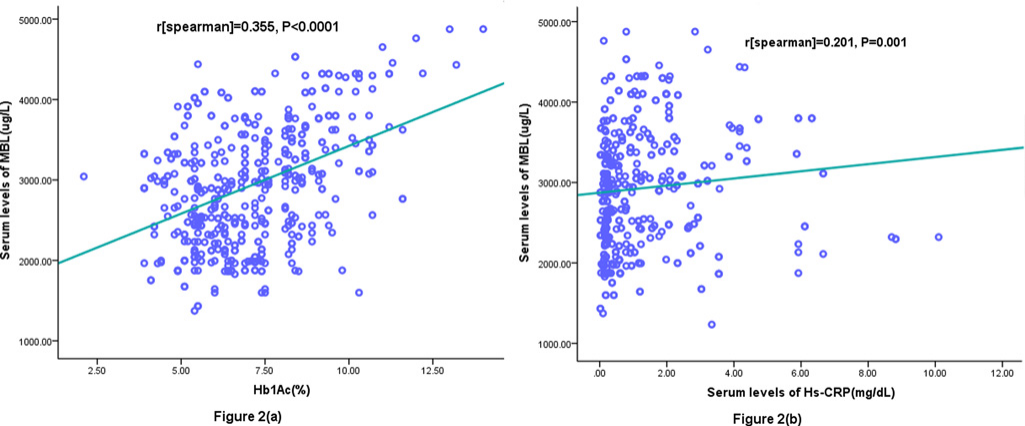
Correlation between the serum MBL levels and other factors (a) Correlation between the serum MBL levels and HbA1c; (b) Correlation between the serum MBL levels and Hs-CRP.

We found that serum MBL levels were significantly higher in diabetes as compared to normal cases [2855(IQR, 2540–3376)ug/l and 875(IQR, 678–992) ug/l, respectively; P<0.0001]. The results indicated that the serum MBL levels were significantly higher in diabetes with DN as compared to with persistent normoalbuminuria [3325(IQR, 2983–3760)ug/l and 2470(IQR, 2105–2942) ug/l, respectively; P<0.0001; Fig. 1]. Serum MBL levels increased with worse of diabetes control as defined by the HbA1c level (Fig. 2a). There was a modest positive correlation between levels of MBL and HbA1c (r = 0.355, P<0.0001) or when the DN and normoalbuminuria groups were analyzed separately (*r* = 0.379, P<0.0001 and *r* = 0.208, P = 0.001; respectively). Similarly, Serum MBL levels increased with severity of DN as defined by the UAE level, and MBL concentrations were positively correlated with UAE (r = 0.215; *P*<0.001). In addition, there was a significant, albeit weak, positive correlation between MBL concentrations and Hs-CRP in the entire study group (*r* = 0.201, *P* = 0.001; Fig. 2b) or when the DN groups were analyzed separately (*r* = 0.256, *P*< 0.0001). There were no significant sex differences, age, creatinine, duration of diabetes, or daily insulin dose.

### MBL and DN

In univariate logistic regression analysis, we calculated the odds ratio (OR) of MBL levels as compared with other factors as presented in Table 2. With an unadjusted OR of 1,002 (95% CI, 1.001–1.002; P<0.0001), MBL had a strong association with DN. In multivariate analysis, after adjusting for all other significant predictors, MBL remained can be seen as an independent DN indictor with an adjusted OR of 1.001 (95% CI, 1.001–1.002; P<0.0001).

**Table 2:** Univariate and multivariate logistic regression analysis for DN

Indictor: DN	Univariate Analysis			Multivariate Analysis		
	OR ^a^	95% CI ^a^	*P*	OR ^a^	95% CI ^a^	*P*
MBL	1.002	1.001–1.002	< 0.0001	1.001	1.001–1.002	<0.0001
MBL(≥2950ug/L)	12.18	4.17–35.08	< 0.0001	7.55	3.44–19.04	<0.0001
Male sex	1.22	1.11–1.35	0.003	1.15	1.06–1.29	0.009
HbA1c	1.09	1.03–1.21	< 0.001	1.05	1.01–1.16	< 0.001
Hs-CRP	1.11	1.05–1.19	< 0.001	1.08	1.03–1.18	< 0.001
Creatinine	1.55	1.30–1.76	< 0.001	1.31	1.10–1.48	0.003
Systolic BP	1.18	1.10–1.32	0.006	1.16	1.05–1.36	0.009

^a^ Note that the odds ratio corresponds to a unit increase in the explanatory variable.

OR, odds ratio; CI, confidence interval; Hs-CRP, High-sensitivity- C-reactive protein; HbA1c, hemoglobin A1c; DN, diabetic nephropathy

Based on the ROC curve, the optimal cutoff value of serum MBL levels as an indicator for diagnosis of DN was projected to be 2950ug/L, which yielded a sensitivity of 77.2 % and a specificity of 80.8%, with the area under the curve at 0.809 (95%CI, 0.769–0.848). With an AUC of 0.809, MBL showed a significantly greater discriminatory ability as compared with Hs-CRP (AUC, 0.67; 95% CI, 0.59–0.78; P = 0.006), HbA1c (AUC, 0.77; 95% CI, 0.69–0.88; P<0.001) and creatinine (AUC, 0.69; 95% CI, 0.61–0.76; P<0.001). Further, in our study, we found that an increased diagnosis ability of DN was associated with MBL levels≥2950ug/L (unadjusted OR 12.18, 95% CI: 4.17–35.08). This relationship was confirmed in the dose-response model. In multivariate analysis, there was an increased diagnosis ability of DN associated with MBL levels≥2950ug/L (OR 7.55, 95% CI: 3.44–19.04; P<0.0001) after adjusting for above possible confounders (Table 2). In addition, male sex, HbA1c, Hs-CRP, creatinine and systolic BP were also can be seen as DN indictors in multivariate analysis (Table 2).

### Discussions

Diabetic nephropathy affects approximately one third of people with type 1 or type 2 diabetes mellitus [12]. As the total number of people with diabetes is projected to increase substantially to 2050, the prevalence of diabetic nephropathy will rise dramatically, with concomitant increase in associated cardiovascular mortality and endstage renal disease. This will produce significant social and economic ramifications, particularly in the developing world, such as China and India.

Mounting evidence suggests that there may be a link between complement activation and the development of diabetic renal complications [13–14]. In this study, we firstly assessed the serum MBL levels with regard to their accuracy to predict DN in patients with T2DM in Chinese sample. There have been several papers in the literature linking MBL and DN complications in T1DM, fewer linking such correction to T2DM and, as far as I could find, none in an ethnic Chinese sample. As such the manuscript adds significantly to the literature, especially as Asian patients with diabetes account for more than 60% of the world’s diabetes population [15].

In our study, we reported that serum MBL levels were significantly higher in patients with DN as compared to persistent normoalbuminuria (P<0.0001). Importantly, for the entire group, when adjusting for other possible risk factors, an elevated MBL level was an independent DN protection factor, and serum MBL levels≥2950ug/L was associated with a 7.55-fold increase in DN, suggesting a possible role of MBL in the pathogenesis of DN complications in diabetes. It could thus be hypothesized that in diabetic patients, high levels of MBL may contribute to the development of nephropathy through aggravated complement activation. Similarly, Hansen et al [5] found that in patients with type 2 diabetes, measurements of MBL alone or in combination with CRP can provide prognostic information on mortality and the development of albuminuria. Further, we found that the serum MBL levels increased with decreasing severity of DN as defined by the UAE.

In previous study, Hansen et al [4] reported that there were no correlations between Hs-CRP and MBL levels (*P* = 0.12), whereas there was a significant, albeit weak, positive correlation between MBL concentrations and HbA1c (*P* = 0.001), UAE(*P* = 0.013) in another study [16]. In our study, we found that serum MBL levels were correlation with HbA1c and UAE, and the relationship between Hs-CRP and MBL was also found. Different information was reported, as there was no correlation between the 2 proteins in previous studies of patients with T1DM [4–5, 17].

That complement activation can be protective and complement proteins such as MBL and C1q can promote anti-inflammatory processes related to apoptotic cell clearance [18–19]. Its protective role notwithstanding, complement may cause or exacerbate inflammatory tissue damage when overactivated or deregulated [20]. The biological mechanism linking MBL with DN is still unclear. Only 30–40% of patients with diabetes mellitus develop overt nephropathy, which suggests that other contributing factors besides the diabetic state are required for the progression of diabetic nephropathy [21]. There are numerous biologically plausible mechanisms by which maternal MBL status could alter risk of DN. Firstly, The distinct difference in MBL levels between diabetic patients with nephropathy and patients with normoalbuminuria was in part attributable to differences in the MBL genotype distribution, indicating that inherited high concentrations of circulating MBL may be a risk factor for diabetic nephropathy [4]. In addition, Ilyas et al [22] reported that there is evidence in vitro of diminished MBL function in the presence of high glucose, such that MBL carbohydrate recognition domains may be less efficient at engaging their targets and driving complement activation in diabetic states with poor glycemic control. Secondly, many lines of evidence, ranging from *in vitro* experiments and pathological examinations to epidemiological studies, show that inflammation is a cardinal pathogenetic mechanism in diabetic nephropathy [23]. Inflammatory cells have all been implicated in the pathogenesis of diabetic nephropathy via increased vascular inflammation and fibrosis [2]. MBL is a slower-reacting and much weaker acute-phase reactant than CRP [24], but it is possible that the MBL level may reflect differences in inflammatory activity. However, the differences in MBL levels between the groups remained statistically significant after correction for differences in Hs-CRP, which indicates that CRP and MBL may carry different types of information as markers of inflammation. In addition, MBL may aggravate local and systemic inflammation through complement activation and modulation of proinflammatory cytokine production [6]. Thirdly, circulating MBL has the ability to effectively initiate inflammation through the enzymatic activation cascades of complement. Complement activation from any cause may thus have more widespread consequences in diabetic patients and contribute to the ongoing inflammation and microvascular and macrovascular complications of diabetes [5]. Fourthly, one study suggested that diabetes patients have more severe oxidative stress than normal persons and higher oxidative stress in diabetic nephropathy than those in patients without complications [25]. MBL could play a role in the progression of DN through oxidative stress. Diabetic mice with severe endothelial dysfunction owing to deficiency of endothelial nitric oxide synthase develop progressive nephropathy and retinopathy similar to the advanced lesions observed in humans with diabetes mellitus [21]. Lastly, MBL-associated enzymes such as MASP-1 and MASP-2 can trigger coagulation cascades that may contribute to tissue damage [26]. Previous studies reported that DN was associated with elevated markers for both coagulation and inflammation [27–28]. Thus, MBL may play a role in the progression of DN through coagulation cascades.

A number of issues have to be taken into account when interpreting the results of the present study. Limitations of our analyses are the relatively small study size and the modest size of the observed effects as well as the unavailability of DNA samples for the analysis of MBL genotypes. Those results should be useful to explain the differences MBL concentration between studies. In addition, without serial measurement of the circulating MBL, this study yielded no data regarding when and how long of MBL was elevated in these patients. Additionally, it should be investigated whether serial MBL testing further improves the risk stratification of these patients. Thirdly, this was only a preliminary study; further studies should investigate whether MBL can help physicians tailor the therapy in view of the relative risk and allocate resources accordingly and whether this strategy might affect DN outcome.

## Conclusions

The present study demonstrated that serum MBL level was an independent risk factor for DN in Chinese patients with T2DM, suggesting a possible role of MBL in the pathogenesis of DN complications in diabetes. We suggested that further studies should be carried out with respect to what was the cause of the increased MBL levels and the role in the pathology of the DN. If it is possible to elucidate this, more intensive efforts could be directed towards the cause, thus hopefully improve the prognosis of these patients.
